# Formation of Australasian tektites from gravity and magnetic indicators

**DOI:** 10.1038/s41598-023-40177-7

**Published:** 2023-08-08

**Authors:** Kurosh Karimi, Gunther Kletetschka, Jiří Mizera, Verena Meier, Vladimír Strunga

**Affiliations:** 1 Institute of Hydrogeology, Engineering Geology and Applied Geophysics, Faculty of Science, Charles University in Prague, Albertov 6, 128 43 Praha 2, Czech Republic; 2 Geophysical Institute, University of Alaska - Fairbanks, 903 N Koyukuk Drive, Fairbanks, AK 99709 USA; 3 Nuclear Physics Institute, Czech Academy of Sciences, Hlavní 130, 250 68 Husinec-Řež, Czech Republic; 4 Institute of Rock Structure and Mechanics, Czech Academy of Sciences, V Holešovičkách 41, 182 09 Praha 8, Czech Republic

**Keywords:** Geology, Geophysics

## Abstract

The parent impact crater of Australasian tektites has not been discovered so far, but a consensus has been accepted on its location in a wider area of Indochina. Recently, an alternative location has been suggested in the Badain Jaran Desert (BJD), Northwest China. Employing gravity and magnetic data derived from satellites, possible presence of an impact structure in BJD is investigated. The gravity parameters include the free air gravity disturbance, its vertical derivative component and total horizontal gradient (THG), strike alignment (SA), and Bouguer anomaly with its first vertical derivative and tilt angle. The magnetic parameters include the anomalous total magnetic field (TMF), its reduced to the pole transformation (RTP), the first vertical derivative of the TMF vertical component (*B*_*zz*_), tilt angle (TA), and logistic total horizontal gradient (LTHG). Both the gravity and magnetic indicators support the presence of the impact structure. Gravity parameters display typical annular gravity highs circumscribing a gravity low. SA analysis reveals preferred parallel directions, implying the susceptibility of special zones to the impact shock waves, both within and beyond the rim. TMF reveals a large magnetic anomaly in the southern part of the proposed crater, and RTP displaces and restricts it further into the rim. *B*_*zz*_ weakens the long wavelength anomalies, amplifies the superficial ones, and separates them horizontally. TA and LTHG delineate the deep-seated and shallow magnetic signals related to the peak and border magnetization, respectively.

## Introduction

Melting of the target materials from the Earth’s surface in an impact of a massive extraterrestrial object can, under conditions not yet clearly understood, lead to formation of natural glasses called “tektites”. Classified as distal ejecta, tektites are transported to a strewn field quite distant from a parent impact structure (crater). Four major tektite strewn fields associated with separate impact structures are recognized: Central European (moldavites), Australasian (indochinites, philippinites, australites, etc.), North American (georgiaites and bediasites), and West African—Ivory Coast (ivorites)^1,2^. Recently, Belize impact glass strewn field has been paired with the Pantasma crater in Nicaragua^3^, although the final assignment is still questioned^1^. There are several other groups of tektite-like glasses of undoubtedly impact origin, which however miss some characteristic tektite properties, e.g., a sufficient distance from the parent crater^1,2^. While three of the main tektite strewn fields have unequivocally been paired with their parent impact craters, the largest one, the Australasian tektite strewn field formed at 0.79 Ma and covering up to a sixth of the Earth’s surface, is still missing discovery of its parent impact structure. Since the paper by Stauffer^4^, the search of a parent crater for Australasian tektites (AAT), has been focused almost solely to Southeast Asia—Indochina, close to the densest occurrence of AAT. The consensus location of the AAT impact in Indochina has been criticized in a series of papers^2,5,6^, the most recent one questioning the location at the Bolaven volcanic field in Southern Laos proposed recently by Sieh et al.^7^. The general criticism has particularly been based on the geochemical inconvenience of AAT composition, pointing to low chemical weathering of source materials, with much more weathered sedimentary targets in Indochina. Also, an Indochina impact location misses analogy with other major tektite strewn fields, where the parent craters are never situated inside the strewn field major area.

**Figure 1:**
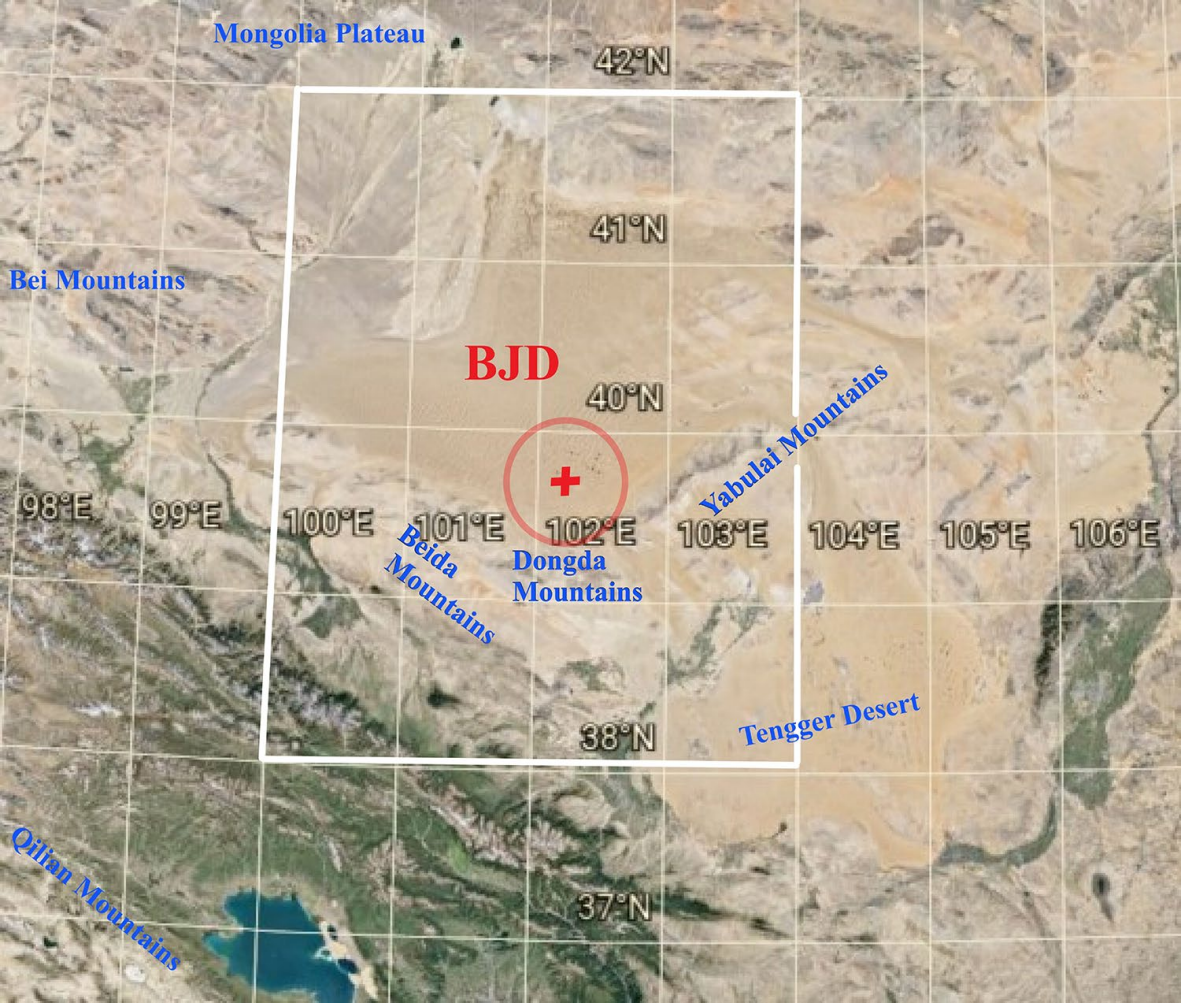
Satellite image of the Badain Jaran Desert (BJD) and its proximity. The hypothetical crater and its center are marked with a red circle and a cross symbol, respectively. The white rectangle indicates the area analyzed in terms of gravity and magnetic parameters.

As an alternative to the criticized consensus location of the AAT impact in Indochina, Mizera et al.^2,5^ proposed a more plausible location in the arid area of Northwest China, most probably in the Badain Jaran Desert (BJD; see the map in Fig. 1), supported by multiple lines of evidence as follows:

(1) Suitability and sufficient supply of pre-impact BJD sediments as an AAT source material, anticipated from close geochemical match between AAT and Chinese loess, and from paleoenvironmental proxies observed in the WEDP02 drill core at the edge of the supposed impact structure. (2) Ideal conditions for crater burial under Holocene sand megadunes, and relatively small ecological consequence of the AAT impact consistent with the oblique impact into a vast desert area surrounded downrange by mountains. (3) Gravity data indicating the existence of anomalous mass within the megadune-lake area in BJD. (4) Specific features of the suggested BJD location possibly attributable to post-impact effects including formation and maintenance of megadunes and lakes, with signs of hydrothermal activity. (5) Presence of possible products of the impactor ablation and other impact-related features in Chinese loess layer recently re-dated to the AAT event. (6) Consistency with principles of distal ejecta ballistic transport, including distribution of various morphological and constitutional AAT types.

Gravity and magnetic anomalies belong to the most apparent geophysical signatures of terrestrial impact structures^8^; see Appendix B in the Supplementary File for more details. Satellite and airborne potential field data serve as useful tools for detecting surface and subsurface large-scale structures on Earth. These datasets have been extensively utilized in numerous studies documented in the literature, showcasing their effectiveness in various applications. For instance, magnetic anomalies associated with the Yallalie impact structure were successfully identified using airborne magnetic data^9^. In a study by Beiki and Pedersen^10^, eigenvector analysis of gravity gradient tensor data provided valuable insights into the strike and depth to the anomalous structures in the Vredefort impact structure. Saada et al.^11^ utilized satellite potential data to investigate the tectonic features of the Red Sea rift. Furthermore, Pham et al.^12^ employed these datasets to detect ridge locations within the Vredefort impact structure. Additional examples include studies by Hamimi et al.^13^, who analyzed potential field data to unveil the geological structures of the Nubian Shield, and Urrutia-Fucugauchi et al.^14^, who conducted a comprehensive airborne potential field analysis of the Chicxulub impact structure. These studies demonstrate the wide-ranging applications and significance of satellite and airborne potential field data in the detection and characterization of Earth's large-scale structures.

Preliminary inspection of the gravity data in the hypothetical impact area in BJD indicated the existence of a roughly circular structure centered at 39.7°N, 102.2°E, within the area of sand megadunes and lakes^5^. The structure was characterized by a pronounced negative gravity anomaly with a diameter ~ 50 km, surrounded by a ~ 100 km rim with a positive gravity anomaly. The observed gravity features were compared with analogous data for the Popigai impact structure, and were consistent with published ground gravity survey data^15^. The present paper continues investigation of the gravity signature of the proposed impact structure in BJD with detailed analysis and interpretation of the free air and Bouguer anomaly functionals both in grid and profile format, and complements it with investigation of magnetic anomaly indicators. The results, interpretation and conclusion presented in this study are not conclusive; Based on the potential field data, we show that the explored area has the characteristics of an impact structure. However, more investigation is needed to confirm this hypothesis.

### Geological setting

The following description has been modified from Mizera et al.^5^, and references therein, mainly^16^. BJD, being the second largest desert in China with an area of 49,000 km^2^, is situated in the western part of the Alxa Plateau and in the southeastern part of the Badain Jaran Basin in northwest/north-central China, Inner Mongolia Autonomous Region, Gansu province (Fig. 1), at an elevation of 900–1500 m a.s.l. Boundaries of BJD are formed by the Mongolian Plateau to the north (N), foothills of Qilian Mts to the south (S), Yabulai (Yabrai) Mts and Zongnai Mts to the east (E), and Bei Mts to the west (W). The Mesozoic and Cenozoic red strata are developed discontinuously in the basin, mainly Cretaceous sandstones and conglomerates, which partially project under the Holocene dune field. The basement is constituted by Proterozoic metamorphic rocks followed by Permian tectonic activity. Several active faults occur south and south-east of BJD—Heli Shan Fault (HLSF), Beida Shan Fault (BSF), Ayouqi Fault (AYQF), and Yabrai Fault (YBF), from W to E (see Fig. 4b)—whose Cenozoic tectono-geomorphic evolution may have affected topographic formation and evolution of mountains and deserts of NW China^17^. A detailed geological map of the area can be found online^18^, for geological maps and geologic profiles across BJD see also^16,19,20^. The hypothetical AAT impact structure is situated in the SE part of BJD, where up to 480 m high and 3–5 km long sand dunes, interspersed with hypo- to hypersaline and alkaline lakes, are found (Fig. 1). BJD belongs to the major source areas of Chinese loess. At the SW edge of the proposed impact area, the Kaxiutata skarn-type iron deposit is located (39.5°N, 101.5°E). The deposit developed near the contact zone between gabbro and Sinian epi-metamorphic rocks. The major mineral is magnetite, minor ones include niccolite, pyrrhotite, safflorite, cobaltite, chalcopyrite, sphalerite, and pyrite^21,22^.

## Results

**Figure 2:**
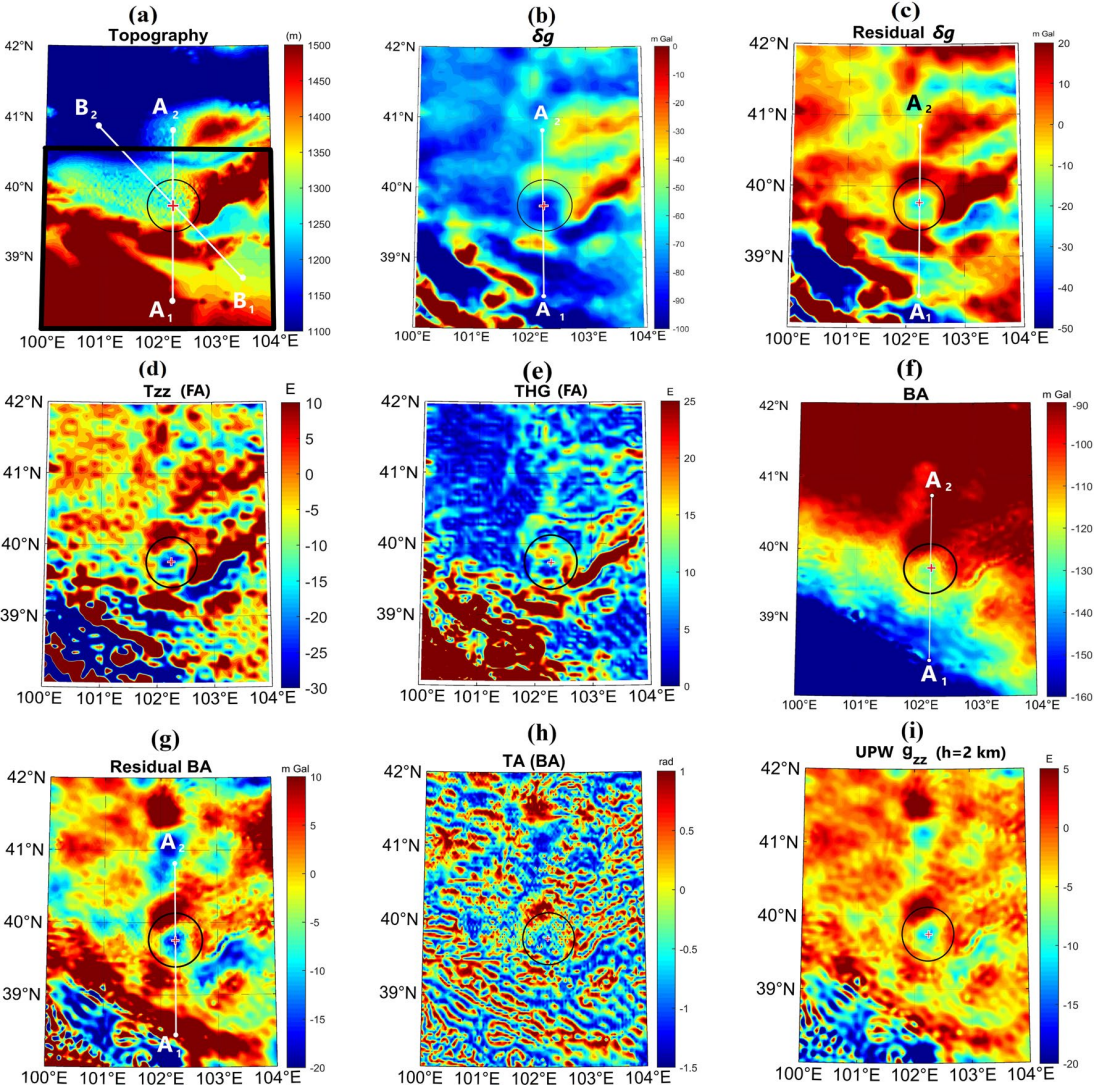
(**a**) Topography. (**b**) Free air gravity disturbance, *δg*. (**c**) Residual *δg*. (**d**) Derivative of *δg* vertical component, *T*_*zz*_. (**e**) Total horizontal gradient, THG. (**f**) Bouguer anomaly, BA. (**g**) Residual BA. (**h**) Tilt angle, TA. (**i**) Continued upward vertical derivative of BA, UPC-*g*_*zz*_. Black circle marks the potential crater, the red cross its center. The A_1_A_2_ and B_1_B_2_ profiles are used in Figs. 3 and 6, respectively. The thick black trapezoid in (**a**) is the exploration area for strike solutions in Fig. 4. The free air (FA) anomalies are shown in (**b**–**e**), the Bouguer type (BA) anomalies in (**f**–**i**).

**Figure 3:**
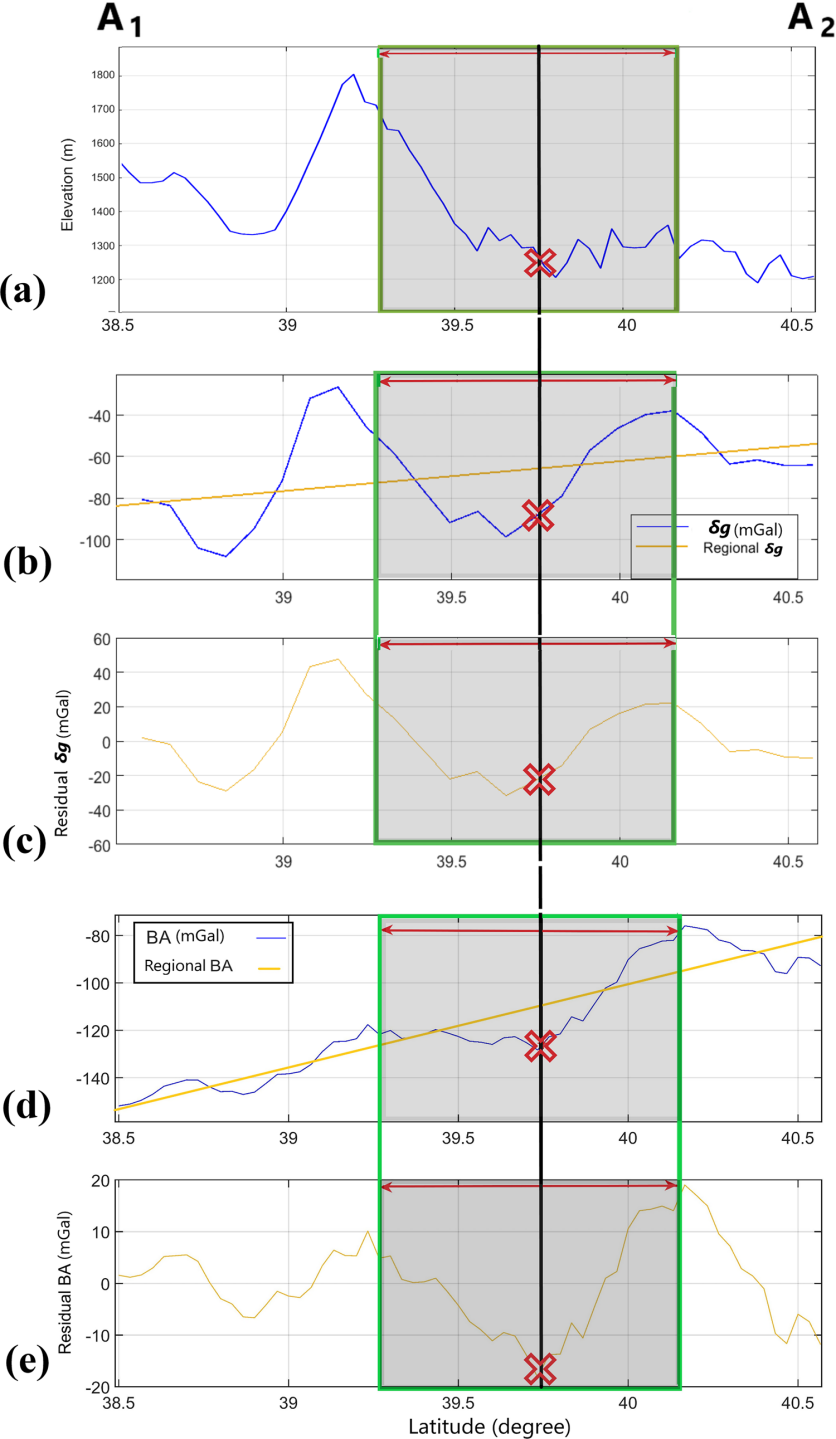
The A_1_A_2_ profile indicated in Fig. 2 for: (**a**) topography; (**b**) δg.; (**c**) residual δg; (**d**) Bouguer anomaly, BA; (**e**) residual BA. The red cross indicates the proposed crater center and the shaded area the horizontal dimension of the crater in the S–N direction.

Figure 2a shows the topography of the area comprising the hypothetical crater location (39.7° N, 102.2° E^5^), shown by a circle and a cross sign in its center. The elevation in the SW of the map is more than 4500 m a.s.l., decreasing to about 1400 m in the middle, and drops to less than 1000 m in the N area. The Beida, Dongda and Yabulai Mts are situated SW, S, and E of the circle, respectively (Fig. 1). The topography variation in the profile A_1_A_2_ (Fig. 2a), going through the crater center from S to N, is presented in Fig. 3a. The elevation from ~ 1700 m a.s.l. over the S rim of the hypothetical crater drops to less than ~ 1300 m on the N rim. The general morphology of the area does not unravel clearly an impact basin. Nevertheless, the gravity parameters indicate a circular pattern with low values in the center and an annular larger value surrounding it. The value in the central zone reaches − 100 mGal for $\delta g$ and − 30 E for *T*_*zz*_. These quantities rise from the center in all directions; at the potential crater rim $\delta g$ averages − 40 mGal on the N and E sides and − 50 mGal in S. The A_1_A_2_ profile indicates two $\delta g$ highs (~ − 40 mGal) on the S and N rims and a low (~ − 100 mGal) in the center (Fig. 3b). Subtraction of the linear regional gravity field (a line in Fig. 3b) from $\delta g$ yields a clearer signature of the residual $\delta g$ attributed to the shallower layers (Figs. 2c, 3c). *T*_*zz*_, the vertical derivative of *δg*, is about zero on average along the rim, with slightly positive values at several locations. The large values of $\delta g$ and *T*_*zz*_ in the S and SW corner of the map are consistent with the high topography in those areas (Fig. 2a,d). The total horizontal gradient (THG; Fig. 2e), sensitive to horizontal variations of the gravity disturbance, demonstrates low values over the crater rim (< 5 mGal m^−1^ on average), and high annular values on the inner flank of the suggested rim. Closer to the crater center, THG is low.

The complete Bouguer anomaly (BA), residual BA, tilt angle (TA), and vertical derivative of BA (*g*_*zz*_) are shown in Fig. 2f–i. The average BA values are ~ − 95 mGal in W, N, and E parts of the potential rim, and ~ − 115 in the S and SW parts. Over the hypothetical cavity, BA decreases to − 125 mGal. The long wavelength regional trend of BA slopes from N to S. The A_1_A_2_ profile on the BA map (Fig. 2f,g) is plotted in Fig. 3d. To consider a BA signature of the shallower layers, we removed a linear regional field and attained a residual BA (Figs. 2g, 3e), where the rim coincides with higher values (~ 10–15 mGal) and the cavity fits a central low (− 18 mGal). The TA parameter is consistent with BA, with emphasis on deep and shallow signals. An annular high and a central low are also observed in this parameter (Fig. 2h). Figure 2i presents the first vertical derivative of BA (*g*_*zz*_). To remove the very shallow noisy signals and emphasize the crystalline bedrock, the *g*_*zz*_ parameter was continued upward (UPC-*g*_*zz*_) to 2 km.

**Figure 4:**
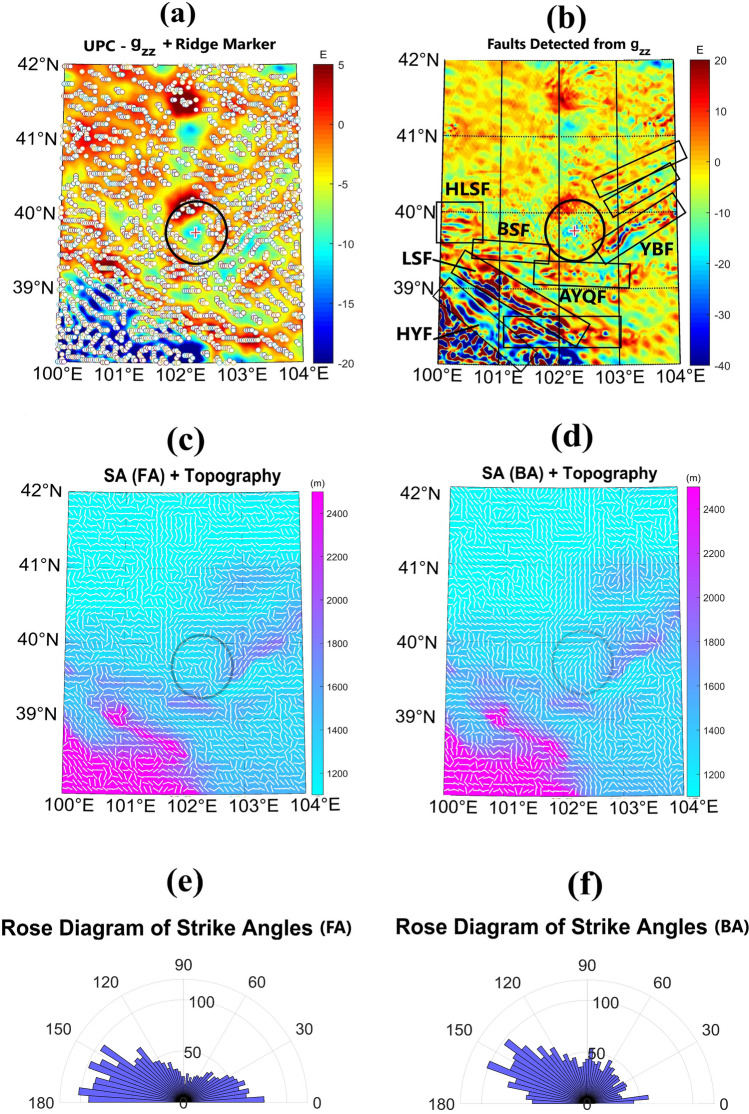
(**a**) Ridge markers (small white circles) fitted on UPC-*g*_*zz*_ map (at h = 2 km). (**b**) Detected faults (delineated with black rectangles; *HLSF* Heli Shan Fault, *BSF* Beida Shan Fault, *AYQF* Ayouqi Fault, *YBF* Yabrai Fault, *LSF* Longshou Shan Fault, *HYF* Haiyuan Fault) with truncating signatures of *g*_*zz*_ parameter. (**c**,**d**) Strike alignment (SA) from free air gravity anomaly (FA) and from Bouguer gravity anomaly (BA), respectively. (**e**,**f**) Rose diagram of strike angle distribution between 0° and 180° with respect to the east axis, with bin width of 4° for FA and for BA, respectively.

To pinpoint the rim structure according to the UPC-*g*_*zz*_ parameter (at h = 2 km), we employed the ridge detection theory by Blakely and Simpson^23^. Figure 4a refers to points where *g*_*zz*_ peaks within a sliding square window with 9 points. The small white circles, obtained for the solutions with significance values N between 2 and 4, characterize the points over the hypothetical crater rim in the intended area. Although the resolution of the data is not high enough to locate all the faults and fractures in the proximity of the proposed crater, several truncated traces in the *g*_*zz*_ map (at h = 0) could be observed in Fig. 4b. These major fault structures are marked by black rectangles in Fig. 4b.

Strike alignment (SA), fitted on topography, for FA and BA types of gravity are plotted in Fig. 4c,d, respectively. The SA points to the exact elongation of the anomalous mass at each data point, whether it is, for example, the wall of the rim or slump, strike slipping or thrust faulting plane. The overall orientation of SA reveals a roughly circular pattern surrounding the rim for both FA and BA gravity maps. This pattern is a typical response of the crater rim. The SA solutions around the crater are consistent with the lineation of topography and faulted structures. The rose diagrams of the strike angles (calculated with reference to the east axis) are plotted in Fig. 4e,f, restricted to 0°–180°. Each bin in the angular histogram indicates a 4° interval. The strike angles between 120° and 180° are dominant.

**Figure 5:**
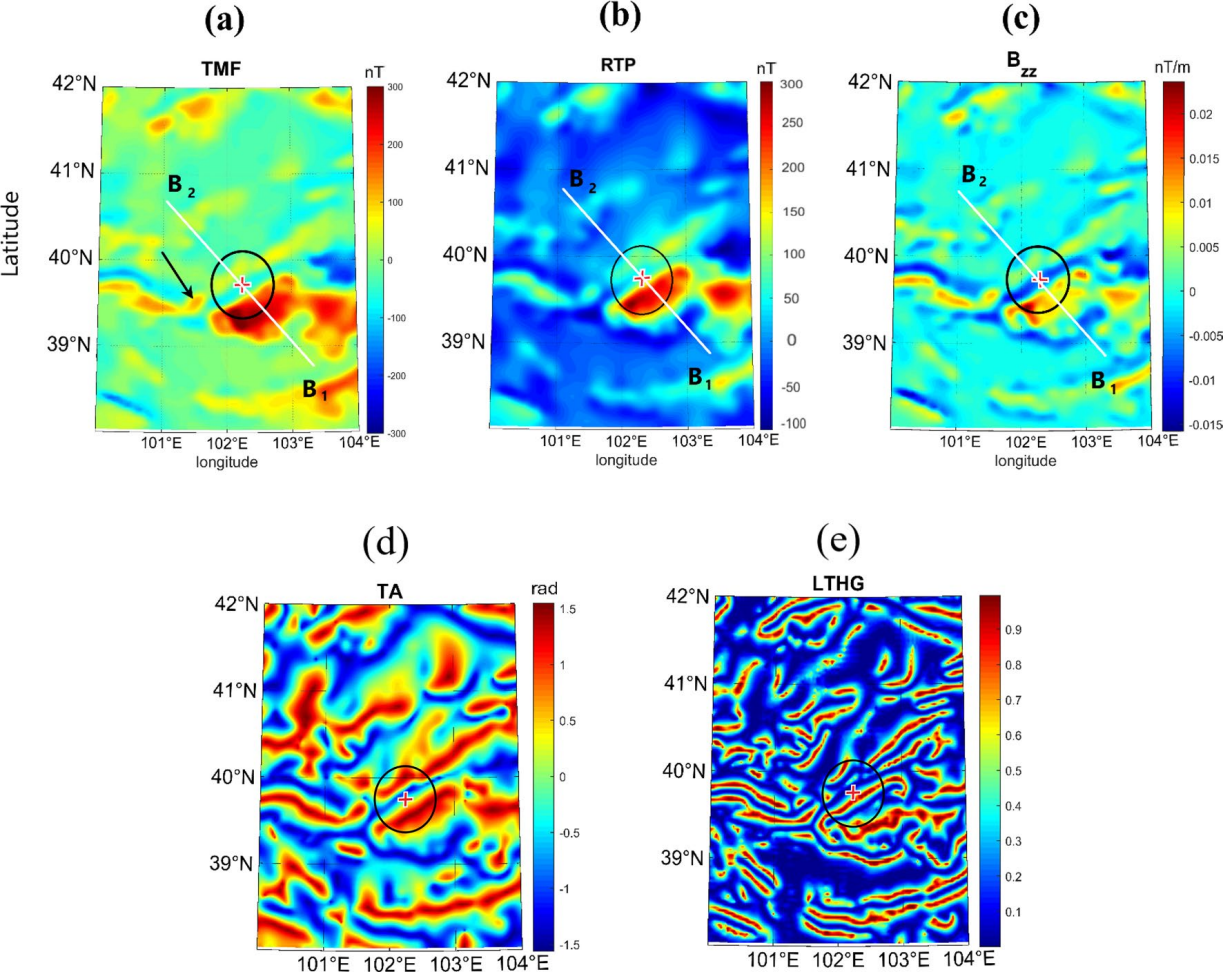
(**a**) Total magnetic field anomaly, TMF. (**b**) Reduced to pole transformation of TMF, RTP. (**c**) Vertical derivative of the vertical component of the TMF, *B*_*zz*_. (**d**) Tilt angle, TA. (**e**) Logistic total horizontal gradient of *B*_*z*_, LTHG. Black circle marks the potential crater, and the cross sign indicates its hypothetical center. The black arrow in (**a**) points to the Kaxiutata iron deposit. B_1_B_2_ profiles are plotted in Fig. 6.

**Figure 6:**
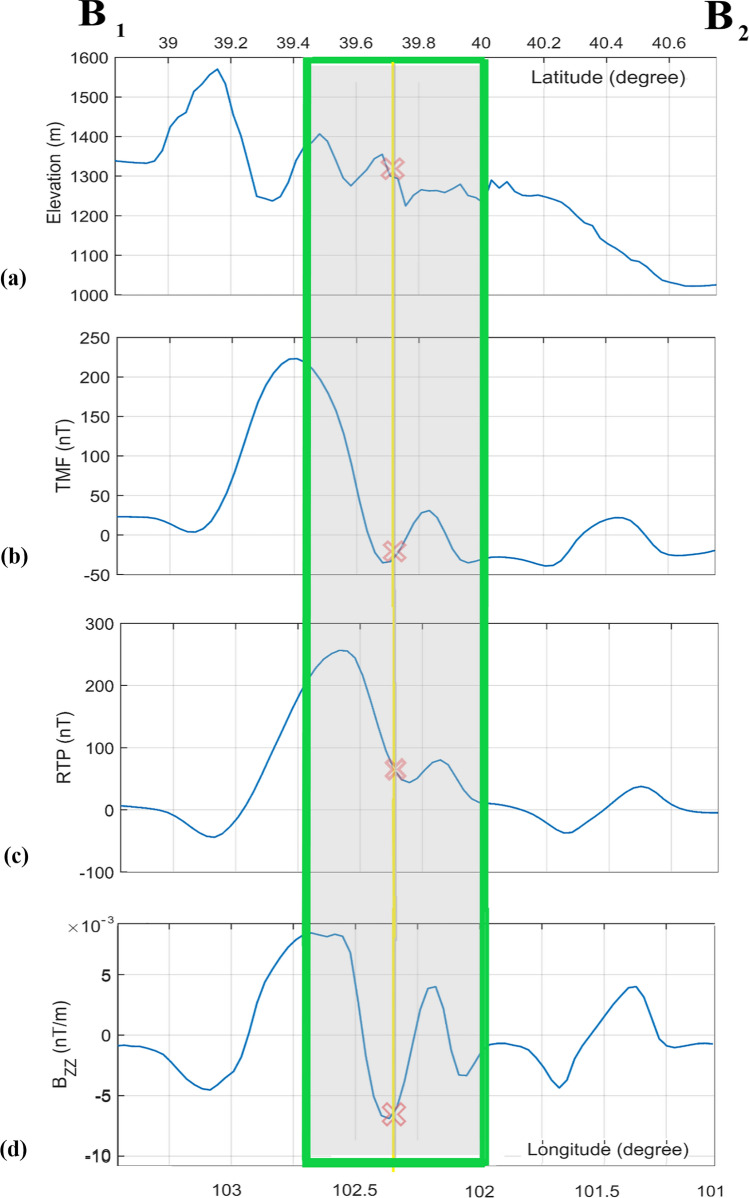
The B_1_B_2_ Profile for: (**a**) topography; (**b**) total magnetic field anomaly, TMF; (**c**) reduction to the pole transformation of TMF, RTP; (**d**) vertical derivative of the vertical component of TMF, *B*_*zz*_. The red cross indicates the proposed crater center and the shaded area the horizontal dimension of the crater in NW–SE direction. The lower and upper axes indicate longitude and latitude, respectively.

Figure 5 shows the magnetic parameter on a grid, and Fig. 6 draws a SE-NW profile (B_1_B_2_, Figs. 2a, 5a–c) over the grid map. The magnetic data indicate a significant positive anomaly in the S and SE parts of the intended area. The anomalous total magnetic field (TMF; Figs. 5a, 6b) reaches its maximum (~ 300 nT) over the hypothetical S rim. There is a mild linear structure of positive value (~ 40 nT on average) trending SW-NE to N of the center cross point. In the center, TMF is about − 30 nT, stretching parallel to its N and S anomalous neighbors. The magnetic pattern partly coincides with the higher topography in S and SW of the crater, whereas this does not hold for the E, NE, and SE districts of the potential crater. The magnetic highs are typically related to ferromagnetic, (ultra)basic–(ultra)mafic rocks, while the magnetic lows indicate acidic–felsic, and most sedimentary rocks. The black arrow in Fig. 5a points to the Kaxiutata iron deposit (see the Geological setting). Figures 6a,b demonstrate the topography and TMF along the B_1_B_2_ profile from SE to NW crossing the potential crater and perpendicular to the magnetic lineation. TMF magnitude on the SE rim is quite high (~ 220 nT), but it amounts to weak signal on the NW rim (~ 30 nT).

Considering the magnetization is entirely induced by the geomagnetic field of the Earth (no remanent magnetization), reduced to pole transformation (RTP) is plotted in Fig. 5b, and its B_1_B_2_ profile in Fig. 6c. As observed in these plots, RTP dominant magnetic response is displaced interior to the crater. *B*_*zz*_ accentuates the shallow (high frequency) linear sources of magnetic properties (Fig. 5c). Figure 6d displays the *B*_*zz*_ parameter along the B_1_B_2_ profile. The magnetic low and highs shown in RTP (Fig. 6c) have been amplified in the *B*_*zz*_ profile, which differentiates the superficially local anomalies. The TA, derived from THG of *B*_*z*_, is almost consistent with *B*_*zz*_. It delineates the surface faults and elevated areas. LTHG map (Fig. 5e) marks the border of the opposing magnetized rock units. The LTHG linear highs coincide with the edges of most of the faults in the S, SW, and W of the impact area.

## Discussion

From the topography map (Fig. 2a), it is clear that the S edge of the suspected parent crater (indicated by a circle) is elevated due to the existence of a mountain range. However, the overall terrain does not show a typical morphology of an impact structure. Since $\delta g$ (Fig. 2b) is a free air type quantity and the gravity induced by the buried impact related terrain affects the field value, $\delta g$ is higher over the rim, and lower over the cavity. The density of material constituting the impact structure and its substrate also affect the magnitude of the field.

The residual $\delta g$ high in the S, E and NE sides of the rim could be partly due to a higher elevation of the terrain. However, on the N, NW and W, the opposite is true, the topography drops, but the residual $\delta g$ is still high, implying the existence of denser rocks in these areas. Comparing the topography, $\delta g$ and residual $\delta g$ of the A_1_A_2_ profile (Fig. 3a–c, respectively) conveys that the large $\delta g$ value of the S rim may be owing to the Dongda Mts, where the larger mass of the mountains is partly compensated by the regional isostasy. Nonetheless, the flexural rigidity of the crust retains a portion of mass uncompensated whereby causing a higher $\delta g$ value. The lack of mass due to excavation, existence of allochthonous and autochthonous breccia, fractured materials, and low density aeolian sand in the upper layer could result in a lower $\delta g$ value within the potential cavity.

Considering that *T*_*zz*_ is amplifying higher frequency signals, the shallowest anomalous signals from each data point are amplified while the deeper ones are attenuated. In other words, the extra mass of the rim and the mass deficiency of the cavity, being closest to the measurement level, should be most boosted. On some points over the candidate rim (Fig. 2d), *T*_*zz*_ is higher, implying that there is an elevated anomalous mass of the rim or uplifted denser bedrock. If the suggested impact crater really exists, according to $\delta g$ and *T*_*zz*_ parameters, its diameter would be ~ 100–120 km (Fig. 2b–d, and shaded area in Fig. 3b,c). Both $\delta g$ and *T*_*zz*_ indicate a narrow band of a higher value in the center from W to NE (see Figs. 2c, 3c). This marginal variation could be due to shallowness of an anomalous mass. If the border of anomalous structure is steep, THG reaches a maximum on the border, with a narrow peak. However, if the margin’s dip is gentle, THG grows from a low, reaches a maximum in the middle of the crater’s flank with a broader peak value, and drops to a low again. Figure 1e demonstrates a circular THG high inside the cavity, concentric with the rim. This pattern refers to the gentle slope of the inner flank of the crater. As the radius increases or decreases, THG drops, indicating the crest of the rim and flat floor of the cavity, respectively.

The BA (Fig. 2f) is virtually consistent with the general BA pattern of the craters with diameter larger than 30 km, where the difference between the average BA values of the potential cavity and the rim reaches a plateau of − 30 mGal^8^. The average difference in our maps is also about – 30 mGal. Lower values of BA in the cavity may be due to post-impact sedimentary infillings. Holocene sediments in the basin mainly consist of thick (up to 500 m) layers of low-density sediments with variable proportion of sand and dust, with the majority of sedimentary infilling being made up of coarse aeolian sand. The desert sand is mainly derived from aeolian reworking of the surrounding mountains and marks the onset of the BJD formation at 1100 ka; approx. 300 ka before the impact^16^. Brecciation and fracturing related to the impact can also contribute to the observed low BA values. Conversely, the higher values of BA over the rim are likely caused by the uplifted denser material (the crystalline bedrock). Additionally, the rise in topography related to the onset of the adjacent orogen gets visible towards the S rim. Since the mountains consist of dense rock compared to low-density basin sediments, the rise in BA values may also be partly related to the location of the crater close to the surrounding mountains on the S rim (Figs. 2f, 3d,e). Note that this interpretation is consistent with the results attained from the ground gravimetry by Yang et al.^15^ to detect topography of bedrock beneath the sand dunes in BJD.

Figure 3d shows the decrease of BA from N to S over the A_1_A_2_ profile. This trend is due to the mountainous zone in the S part of the map (see Figs. 2a, 3e) where the highlands’ root extends deeper into the mantle according to isostasy. The TA parameter has almost the same properties as *g*_*zz*_; the difference is that unlike *g*_*zz*_, accentuating the shallower signals, TA emphasizes the anomalous structures at different depths. A number of faults in the vicinity of the potential crater could be observed from the TA map (Fig. 2g). The UPC-*g*_*zz*_ is another parameter that, ignoring the morphology of the layering, can reveal a circular denser structure, comprising less dense rock units in the center (Fig. 2i).

From the ridge detection theory^23^, we have plotted small white circles in Fig. 4a to show the points where UPC-*g*_*zz*_ (at h = 2 km) is maximum with significance levels of 2, 3, and 4; in other words, each of these circles signifies the *g*_*zz*_ parameter larger than its neighbors in 2, 3 and 4 directions, respectively. The significance value, $N\ge 2,$ gives acceptable results for the ridge detection (Blakely and Simpson, 1986). These circles coincide with the presumed rim of the proposed crater. The *g*_*zz*_ at a zero elevation also has the capability of detecting discontinuities like faults. The faults, shown by black rectangles in Fig. 4b, truncate *g*_*zz*_ with low linear or curvilinear quantities, coinciding with their planes, and parallel highs on either side. The faults lie in the NE, S, SW and W of the crater. Considering the direction of impactite from NW to SE^5^ and strike of the faults close to the rim (NE-SW and W-E), it seems that the crater truncates these faulting trends. That is, the faults run from NE, disappear in the middle (in location of the crater), and then reappear on the W margin of the crater. This could imply the younger age of the crater with respect to the faults in its proximity. Du et al.^17^ reported on the Cenozoic tectono-geomorphic evolution of the Yabrai Fault (YBF) and its impacts on the landscape formation of BJD during the late Quaternary. YBF is characterized by left-lateral strike-slip faulting with normal faulting components neighbouring the E part of the proposed impact structure. A 500 m uplift of the YBF central segment created a topographic barrier separating BJD and the Tengger Desert (see Fig. 1), which may have played a key role in formation of the megadune-lake pattern. Dating of the onset of normal faulting to 5–2 Ma based on very scattered (U-Th)/He data and slip rates obtained from U-Th dating of calcite formation on fault striation and ^10^Be exposure dating of river terraces and alluvial fans, both dated to the Late Pleistocene, is inconsistent with the abrupt change from the desert to lake environment in the Middle Pleistocene. The uplift process may have started much later and was much faster, consistent with the impact into the present megadune-lake area at the foothills of the Yabulai Mts at 0.79 Ma.

Typically, the rim and areas close to the impact crater could be affected by the impact energy discharge. The uplifted deformed rim and developed fracturing, slump and thrust faulting planes within the rim arrange in a roughly circular pattern, showing a high comb factor; the SA solutions seem to be combed in preferred directions (Fig. 4c,d)^24–26^. Additionally, beyond the crater rim, some zones with weaker structures may also be affected by the shock wave of the impact, thereby undergoing more intense deformation, fracturing, schistosity, and accumulation. Consequently, the impact zone would have some areas with parallel SA in its vicinity farther from the crater rim. For example, in the area under question, Fig. 4c,d demonstrate that there are some biased lineaments from 120° to 180° (Fig. 4e,f). These lineaments indicate the topography and fault system trends, which could not be due to the impact, but possibly, affected by it.

The typical magnetic signature in the area is different from the gravity, indicating more complicated behavior of the magnetic property compared with the gravity. The TMF value (~ 300 nT) at the S rim is probably partly due to the mountainous structure of the granitic bedrock, where the measurement distance is smaller (4 km above the reference ellipsoid) (Figs. 2a, 5a). This large magnetic anomaly, however, extends to W, where elevation drops. The shift of RTP towards the center of the potential crater is neither consistent with topography nor with the gravity anomaly. This strengthens the hypothesis in favor of the presence of highly magnetic, but thin and wide layer under the sands of the S part of BJD. This broad anomaly could be a shallow thin melt sheet. Its positive signature implies an induced source of magnetization or re-magnetization at a time when geomagnetic field was of normal polarity, same as today. Stratigraphic positions of microtektite layers associated with the Australasian impact indicate that the impact happened 12–15 ka prior to the Matuyama–Brunhes geomagnetic reversal^27^, at a time when geomagnetic field was about to change its polarity from reverse to normal. The melt sheet must have been above the Curie temperature thousands of years after the impact, and cooled below this temperature after the geomagnetic field underwent a polarity change from reverse to normal. Then the melt sheet acquired a positive remanent magnetization persisting to the present day. Such a scenario is consistent with models of cooling time of impact melts^28,29^, as well as with the signs of hydrothermal activity observed in the proposed impact area in BJD. Formation of sublacustrine carbonate spring mounds in BJD lakes resembles the postimpact hydrothermal activity known from the Ries impact structure^5^. Recently reported “warm island effect” observed in the BJD megadune-lake area^30^ may also support our hypothesis.

The positive and negative bands of *B*_*zz*_ strengthen the signals from to the most superficial or smallest scale anomalies. The stripping *B*_*zz*_ pattern throughout the whole map (Fig. 5c) outlines the extent of a magnetic carrier formation and suggests directionality from NW to SE. A narrow band of negative *B*_*zz*_ values around the large magnetic anomaly S of the crater (Fig. 5c) indicates the location where most of the momentum of the impactor was delivered and thus where most shock melting and vaporization took place. Unlike *B*_*zz*_ which focuses on small or shallow structures, TA boosts the strongest signals from both deep and shallow structures (Fig. 5d). The TA marks some of the faults and crystalline rocks with distinct magnetic signatures. Except the Yabrai Fault (YBF), all other faults, and the boundary to the likely melt sheet inside the cavity detected from RTP, could be marked by the LTHG map (Fig. 5e).

## Conclusions

We provide analysis and interpretation of satellite magnetic and gravity data in the SE part of the Badain Jaran Desert (BJD), Northwest China, to seek for a potential zone for finding the parent impact crater of Australasian tektites (AAT). Although differently, both gravity and magnetic data represent supportive evidence for the presence of a suitable large crater on the southern margin of BJD, buried under sand megadunes. The dynamic nature of the Earth’s interior and atmosphere tend to eliminate the crater’s trace, thus it is not surprising that the morphology of the area does not give any information in this regard.

From the gravity disturbance ($\delta g$) and Bouguer anomaly (BA) it can be concluded that there is a high density ring-like structure encircling a low density cavity. The average difference between BA values over the likely cavity and rim (~ 30 mGal) supports the probability of the existence of a crater in the area. Both $\delta g$ and BA highs over the rim may be owing to the impact related uplifted crystalline bedrock. On the contrary, lower $\delta g$ and BA in the center could result from lack of mass due to excavation, brecciated and fractured infill materials, and Holocene low-density sediments (sand and dust).

The high frequency amplifying filters, *T*_*zz*_ and *g*_*zz*_, reveal the shallow masses, the former with topography and the latter without it. A rim structure and cavity with larger and smaller densities, respectively, being the most surficial features, are detected by these two parameters. The tilt angle (TA) and *g*_*zz*_ exhibit the rim structure, the cavity, and several faults surrounding the crater. From the *g*_*zz*_ signature, the faults running from NE side of the crater to its W seem to have been truncated by the impact.

Due to the amount of energy discharged within the impact area, the rim structure is deformed and fractured. The strike alignment (SA) solutions could reveal the lineaments of the fractures and accumulation of material, which are along the perimeter of the rim. Farther from the rim, some zones are affected more by the impact energy, and the structural weakness prompts these zones to develop parallel SA and high comb factors.

The most prominent magnetic anomaly is situated in the S and SE of the potential crater, probably downrange the trajectory of the impactor motion from NW to SE. Assuming that the magnetization is solely induced by the ambient magnetic field, the reduced to the pole (RTP) map shifts this anomaly further into the crater, reinforcing its association with the crater itself. A comparison between the RTP and BA maps suggests that the magnetic anomaly within the crater is not likely to be thick or deep but rather thin, shallow, and extensive. This is supported by the absence of a corresponding significant mass anomaly, containing ferromagnetic material, in the BA map. The positive sign of the magnetic anomaly near the crater indicates induced magnetization or remagnetization of the large melt pool during a normal polarity phase.

While *B*_*zz*_ accentuates and differentiates the shallow sources of magnetism, TA delineates the deep-seated and shallow structures with distinct magnetic properties like faults and mountains. LTHG, on the other hand, marks the borders where the magnetization changes. Therefore, TA and LTHG can isolate the anomalous RTP, suggesting an existence of a large rock body shock-melted by the impact.

To summarize, the gravity and magnetic data support the assumption that the AAT parent crater may be present in the megadune-lake area of BJD.

## Data and methodology

In this study, we employed the combined free air gravity field model EIGEN-6C4^31^, and a Bouguer model derived from it^32^, the EMAG2 magnetic model^33^, and the ETOPO1 topography model^34^. We derive free air gravity disturbance $(\delta g)$ and its vertical component derivative (*T*_*zz*_), strike angle (SA), Bouguer anomaly (BA), tilt angle (TA) and the first vertical derivative of BA (*g*_*zz*_), total magnetic field anomaly (TMF), reduction to the pole transformation (RTP), the first vertical derivative of the vertical component of TMF (*B*_*zz*_), and TA and logistic total horizontal gradient (LTHG) of RTP. The derivatives are calculated through the fast Fourier transform^35–37^. For details see Appendix A in the Supplementary file.

### Supplementary Information

Supplementary Information.

## Data Availability

The data are available through Mendeley Data: https://doi.org/10.17632/g7jpg668wf.1.
