# Supplementary material for: Formation of Australasian tektites from gravity and magnetic indicators
Source: Sci Rep. 2023 Aug 8;13:12868. doi: 10.1038/s41598-023-40177-7 (PMC10409792; doi:10.1038/s41598-023-40177-7)
Supplement: Supplementary file 1 — Supplementary Information. [file 41598_2023_40177_MOESM1_ESM.docx]

**Formation of Australasian tektites from gravity and magnetic indicators**

**Supplementary information**

**Kurosh Karimi^1^*, Gunther Kletetschka^1,2^, Jiří Mizera^3,4^, Verena Meier^1^, Vladimír Strunga^3^**

^1^ Institute of Hydrogeology, Engineering Geology and Applied Geophysics, Faculty of Science, Charles University in Prague, Albertov 6, 128 43 Praha 2, Czech Republic.

^2^ Geophysical Institute, University of Alaska - Fairbanks, AK 99709 Fairbanks 903 N Koyukuk Drive, AK, USA

^3^ Czech Academy of Sciences, Nuclear Physics Institute, Hlavní 130, 250 68 Husinec-Řež, Czech Republic
^4^ Czech Academy of Sciences, Institute of Rock Structure and Mechanics, V Holešovičkách 41, 182 09 Praha 8, Czech Republic

Corresponding author: Kurosh Karimi (karimik@natur.cuni.cz)

**Appendix A**

**Gravity gradient tensor and its properties**

The gravity gradient tensor (GGT) or Marussi tensor is a multichannel dataset represented as a symmetric matrix with five independent components:

$\boldsymbol{\Gamma}= \nabla(\boldsymbol{\nabla}T)=\left[ \begin{matrix} \Gamma_{ii} & \Gamma_{ij} & \Gamma_{ik} \\ \Gamma_{ji} & \Gamma_{jj} & \Gamma_{jk} \\ \Gamma_{ki} & \Gamma_{kj} & \Gamma_{kk} \end{matrix} \right]$ (1)

where *T* is the disturbing potential, subscripts *ij* are two orthogonal components of the coordinate system (e.g., Cartesian), where each of “*i*” and “*j*” are *x*, *y*, *z*. The second derivative components of the gravity potential are $\Gamma_{ij}= \frac{\partial}{\partial i}\left( \frac{\partial T}{\partial j} \right)= \frac{\partial^{2}T}{\partial i \partial j}=\Gamma_{ji}$.

There are five independent components in equation (1) for two reasons: (1) In a free source condition, i.e., the measurement is done out of the gravitational source, the Laplace equation holds, i.e., $\nabla^{2} T=0and\Gamma_{kk}=-(\Gamma_{ii}+\Gamma_{jj})$, and (2) $\Gamma$ is symmetric ($\Gamma_{ik}=\Gamma_{ki}, \Gamma_{ij}=\Gamma_{ji}, and \Gamma_{jk}=\Gamma_{kj}$). The disturbing potential is [1]:

$T\left( r,\varphi,\lambda\right)=\frac{GM}{r}\sum_{l=2}^{\infty} \sum_{m=0}^{l} ( {\frac{R_{p}}{r})}^{l}\left\{ C_{lm}^{'}cos m\lambda+S_{lm}sin m\lambda\right\} P_{lm}(sin \varphi)$ (2)

where *G* and *M* are the universal gravity constant and mass of the planet, respectively. R*_p_* is the radius of the planet at the equator, *r* is the radial distance of a point outside the planet where *T* (disturbing potential) is calculated, $P_{lm}(sin \varphi)$ is a Legendre polynomial function, *l* and *m* are the degree and order of the harmonic expansion, respectively, and $\lambda\mathrm{and} \varphi$ are the geocentric longitude and latitude. $C_{lm}^{'}$and $S_{lm}$ are normalized Stokes parameters (harmonic geopotential coefficients), where $C_{lm}^{'}=C_{lm}-C_{lm}^{el}$, with $C_{lm}^{el}$ being the coefficient of the reference ellipsoid.

The spherical approximation of the gravity disturbance is [1]:

$\delta g=-\frac{\partial T}{\partial r}$ (3)

**Tensor components** **in Cartesian coordinate system**

In a Cartesian coordinate system, several of the GGT components relate to a crustal material-specific density distribution, and have the following properties:

$\Gamma_{zz}$ is the first vertical derivative of the vertical gradient of the disturbing potential. $\Gamma_{zz}$is, in essence, a high pass filter with a strengthening property of the signals from shallow structures. $\Gamma_{zz}$ is the strongest signal to noise among all GGT components [2].

$\Gamma_{xz}, \Gamma_{yz}$ are the horizontal derivatives of the vertical gradient of the disturbing potential in the $\hat{i}$ and $\hat{j}$ directions. They delineate the edges of the anomalous density structures or contact areas as$\mathrm{THG} (\mathbf{T}\mathrm{otal}\mathbf{H}\mathrm{orizontal}\mathbf{G}radient)=\sqrt{{\Gamma_{xz}}^{2}+{\Gamma_{yz}}^{2}}$, which is invariant about the $z$ axis. When the contact dip between contrasting density volumes is vertical, THG indicates a clear edge structure, while in the case of gentle slopes, the magnitude of this parameter decreases towards the noise level [2].

$\Gamma_{xx}, \Gamma_{yy}$ are two horizontal derivatives of the horizontal gradients of the disturbing potential. Through Laplace equation, $\Gamma_{xx}+\Gamma_{yy}=-\Gamma_{zz}$.

$\Gamma_{xy}\mathrm{or}\Gamma_{yx}$ is a horizontal derivative of the horizontal components. The derivative and the component are perpendicular. $\Gamma_{xy}$can pinpoint the corners of the underground volumes of contrasting density.

**Invariants**

Tensor $\boldsymbol{\Gamma}$ has three invariants *I_0_, I_1_*, and *I_2_*, meaning that under any coordinate rotation, their values do not change [3]:

$I_{0}=Trace\left( \Gamma\right)=\sum_{i=1}^{3} \Gamma_{ii}=0$ (4)

$I_{1}=\frac{1}{2}\left( (Trace {\left( \Gamma\right))}^{2}-Trace(\Gamma^{2}) \right)=\Gamma_{ii}\Gamma_{jj}+\Gamma_{ii}\Gamma_{kk}+\Gamma_{jj}\Gamma_{kk}-{\Gamma_{ij}}^{2}-{\Gamma_{jk}}^{2}-{\Gamma_{ik}}^{2}$ (5)

$I_{2}=det\left( \Gamma\right)=\Gamma_{ii}\left( \Gamma_{jj}\Gamma_{kk}- \Gamma_{jk}\Gamma_{kj} \right)+\Gamma_{ij}\left( \Gamma_{jk}\Gamma_{ki} - \Gamma_{ji}\Gamma_{kk} \right)+\Gamma_{ik}\left( \Gamma_{ji}\Gamma_{kj} - \Gamma_{jj}\Gamma_{ki} \right)$ (6)

Since $\boldsymbol{\Gamma}$ is a symmetric matrix, its eigenvectors and eigenvalues should be perpendicular and real, respectively. From this condition, it follows that:

$0\leq I=-\frac{{{(I}_{2}/2)}^{2}}{{{(I}_{1}/2)}^{3}}\leq1$ (7)

This implies that *I*_1_ < 0 in any case.

*I*_1_ and *I*_2_ are two high pass filters amplifying the sources near the measurement point (surface) with units of s^-4^ and s^-6^, respectively. For example, for a point source $I_{1}=-3\frac{{(Gm)}^{2}}{r^{6}}$ and $I_{2}=-2\frac{{(Gm)}^{3}}{r^{9}}$ [3]. Compared with $T_{zz}=2\frac{Gm}{r^{2}}$, the strength of the filters in passing the high frequency signals is as follows:

*I*_2_ *> I*_1_ *> T_zz_*. This means that, increasing the distance, the *I*_2_ weakens the deep anomalous sources faster than *I*_1_, and *I*_1_ faster than *T_zz_*. *I*_1_ quantity is different from the other two in that this parameter cannot distinguish the negative sources from the positive ones. In other words, both positive and negative signals are boosted without showing their signs (*I*_1_ is always negative). So, this feature could be regarded as a disadvantage. On the other hand, *T_zz_* and *I*_2_ maintain the signs of the anomalies. These high pass filters should be treated with caution because they are more susceptible to noise as the power of “*r*” rises.

*I* can be called a “dimensionality indicator” [4] whose “zero” value shows a pure 2D distributed density, and value approaching “one” signifies a density distributed in 3D. A pure 2D body is a body in which one horizontal dimension goes to physical infinity and becomes much larger than the other horizontal and vertical dimensions. In a pure 3D mass, the two horizontal dimensions of the body are almost the same size; the vertical dimension could be smaller or larger. It should be noted, however, that although a causative body is 3D, the determinant of GGT (*I*_2_*)* and *I* might be zero at some points of the measurement plane. Therefore, the zero value of *I* for the 2D state is a necessary condition but not sufficient. In other words, a 2D mass has a zero value of *I*, but a zero value of *I* does not necessarily mean that the mass is 2-dimensional. In contrast, *I=*1 always signifies 3-dimensionality.

There is no distinct criterion for separation between 2- and 3-dimensionality. Some consider *I=*0.3 [5] and others consider *I=*0.5 [4] as a threshold for differentiating 2D from 3D bodies. The closer *I* is to unity, the closer the mass is to a pure 3D body like a sphere. A closer amount to zero could represent 2‑dimensionality for cases when the determinant of GGT is zero. In a case where a 2D body is along the *x* axis, the first row and column of $\boldsymbol{\Gamma}$ are near zero, and *I*_2_ = *I* ~0.

$$\boldsymbol{\Gamma}=\left[ \begin{matrix} 0 & 0 & 0 \\ 0 & \boldsymbol{\Gamma}_{yy} & \boldsymbol{\Gamma}_{yz} \\ 0 & \boldsymbol{\Gamma}_{yz} & \boldsymbol{\Gamma}_{zz} \end{matrix} \right]$$

Note that 3- or 2-dimensionality also depends on the measurement point distance from the underground causative body as well as the grid data points. For instance, an anomalous structure such as the oceanic-continental plate boundary might seem 2D from a far measurement point (for example, at h=50 km from the reference ellipsoid), while it appears 3D if we conduct airborne gravimetry at h=2 km with a grid network of 100 m × 100 m.

**Strike alignment (strike direction)**

Strike alignment is a direction along which the gravitational response of a geological construct is constant.

Consider a long horizontal cylinder with strike alignment along *x* axis, the coordinate system should be rotated around *y* axis in such an angle that in a least square sense, the rotated axis (*x’*) fits the strike direction of the body. This rotation angle yields the strike direction of the body [3]. In the rotated coordinate system (*i’*, *j’*, *k’*):

$$\boldsymbol{\Gamma'}=\left[ \begin{matrix} {\Gamma'}_{ii} & {\Gamma'}_{ij} & {\Gamma'}_{ik} \\ {\Gamma'}_{ij} & {\Gamma'}_{jj} & {\Gamma'}_{jk} \\ {\Gamma'}_{ik} & {\Gamma'}_{jk} & {\Gamma'}_{kk} \end{matrix} \right]$$

To find the strike direction, $\theta_{s},$the objective function, $Q={{\Gamma^{'}}_{ii}}^{2}+{{\Gamma^{'}}_{ij}}^{2}+{{\Gamma'}_{ik}}^{2}$, should be minimal with respect to $\theta_{s}$ (modified from [3]):

$$\frac{\partial Q}{\partial\theta_{s}}=\frac{\partial}{\partial\theta_{s}}[{{\Gamma^{'}}_{ii}}^{2}+{{\Gamma^{'}}_{ij}}^{2}+{{\Gamma'}_{ik}}^{2}]=0$$

$\theta_{s}=\frac{1}{2}\left\{ {tan}^{-1}(2 \frac{\Gamma_{ij}\left( \Gamma_{ii}+\Gamma_{jj} \right)+ \Gamma_{ik}\Gamma_{jk}}{{\Gamma_{ii}}^{2}-{\Gamma_{jj}}^{2}+{\Gamma_{ik}}^{2}-{\Gamma_{jk}}^{2}}) \right\}=\frac{1}{2}\left\{ {tan}^{-1}(2 \frac{{-\Gamma}_{ij}\Gamma_{kk}+ \Gamma_{ik}\Gamma_{jk}}{{\Gamma_{ik}}^{2}-{\Gamma_{jk}}^{2}+\Gamma_{kk}(\left( \Gamma_{ii}-\Gamma_{jj} \right))}) \right\}$ (8)

Note that $\theta_{s}$ could be computed within a multiple of $\frac{\pi}{2}$, i.e., equation (8) gives an extreme value for $Q$. Thus, $Q\left( \theta_{s} \right) and Q(\theta_{s}+\frac{\pi}{2}$) at each data point should be calculated, and the minimum value gives the true strike direction, provided that *I* is small. The strike direction could also be derived from the direction of the eigenvectors corresponding to the minimum eigenvalues of the GGT tensor [4]. It is worth mentioning that the intended coordinate system in [3] differs from the coordinate system that is utilized in our work. Consequently, equation (8) should be modified with respect to the adopted reference frame. For the east, north, up (ENU) reference frame:

$\tan\left( 2\theta_{s} \right)=2 \frac{\Gamma_{en}\left( \Gamma_{ee}+\Gamma_{nn} \right)+ \Gamma_{ez}\Gamma_{nz}}{{\Gamma_{ee}}^{2}-{\Gamma_{nn}}^{2}+{\Gamma_{ez}}^{2}-{\Gamma_{nz}}^{2}}$ , where $\theta_{s}$ is measured with respect to the east axis.

**Tilt Angle**

In contrast to the components of GGT that amplify the shallow signals, tile angle could detect the signals from different depths. It is defined as [6]:

${TA=tan}^{-1}\left( \frac{\Gamma_{zz}}{\mathrm{THG}} \right)={tan}^{-1}\left( \frac{\Gamma_{zz}}{\sqrt{{\Gamma_{xz}}^{2}+{\Gamma_{yz}}^{2}}} \right)$ (9)

**Calculation of Bouguer gravity gradient tensor**

The Bouguer anomaly (*g_z_*) and total magnetic field (TMF) data at our disposal are in columnar format (longitude, latitude, *g_z_*, TMF) in Cartesian coordinate system. To calculate the directional derivatives (Marussi tensor) of *g_z_*, we take advantage of Hilbert transform and Fourier domain. Firstly, from *g_z_*, the horizontal components of Bouguer type gravity anomaly (*g_x_* and *g_y_*) are calculated. Then, the first derivatives of these three components are computed in different directions. The horizontal and vertical derivatives of Bouguer potential, *U*, are Hilbert transforming pairs^8^:

$\left\{ \begin{aligned} \frac{\partial U}{\partial z}\mathcal{=H}\left( \nabla_{H}U \right) \\ \mathcal{F}\left( \frac{\partial U}{\partial z} \right)=-i \frac{k_{x}}{|k|}\mathcal{F}\left( \frac{\partial U}{\partial x} \right)-i \frac{k_{y}}{|k|}\mathcal{F}\left( \frac{\partial U}{\partial y} \right) \end{aligned} \right.$ (10)

where $\mathcal{H}$is Hilbert transform operator, $\mathcal{F}$ is Fourier transform, *k_x_* and *k_y_* are wave numbers in $\hat{i}$ and $\hat{j}$ directions, $\left| k \right|=\sqrt{{k_{x}}^{2}+{k_{y}}^{2}},$ ‘*i*’ is an imaginary unit, and $\nabla_{H}=\frac{\partial}{\partial x}\hat{i}+\frac{\partial}{\partial y}\hat{j}$

Using inverse of equation (10) we have:

$\left\{ \begin{aligned} g_{x}=\frac{\partial U}{\partial x}= \mathcal{F}^{-1}\left( i \frac{k_{x}}{|k|}\frac{\partial U}{\partial z} \right) \\ g_{y}=\frac{\partial U}{\partial y}= \mathcal{F}^{-1}\left( i \frac{k_{y}}{|k|}\frac{\partial U}{\partial z} \right) \end{aligned} \right.$ (11)

If we repeat the same process of relation (10) for *g_x_*, *g_y_*, and *g_z_*, the directional derivatives of $\vec{g}$ are attained:

$g_{mn}=\mathcal{F}^{-1}[ik_{m}\mathcal{(F(}g_{n}))]$ (12)

where $\left\{ \begin{aligned} m=\hat{i}, \hat{j}, \hat{k} \\ n=\hat{i}, \hat{j}, \hat{k} \end{aligned} \right.$ are the unit vectors in three orthogonal directions in the Cartesian coordinate system.

Now, components of the Bouguer type Marussi tensor could be written as:

$\mathcal{g}=\left[ \begin{matrix} g_{ii} & g_{ij} & g_{ik} \\ g_{ji} & g_{jj} & g_{jk} \\ g_{ki} & g_{kj} & g_{kk} \end{matrix} \right]$ (13)

**Calculation of vertical component of the magnetic anomaly and its vertical derivative**

To calculate the vertical derivative of vertical component of the magnetic anomaly ($B_{zz}$), we first need to find the vertical component of magnetic anomaly (*B_z_*) from TMF. We follow derivations from [7] here. The relation between vertical component of the magnetic anomaly and TMF in the Fourier domain is:

$\mathcal{F}\left( B_{z} \right)\mathcal{= F}\left( \psi_{z} \right)\mathcal{F}\left( \mathrm{TMF} \right)$ (14)

where $\mathcal{F}\left( \psi_{z} \right)=\frac{1}{\theta_{f}}=\frac{1}{f_{z}+i\frac{f_{x}k_{x}+f_{y}k_{x}}{|k|}}$, $\mathcal{F}\left( \psi_{z} \right)$ is the vertical component operator.

$\theta_{f}=f_{z}+i\frac{f_{x}k_{x}+f_{y}k_{x}}{|k|}$ , is the phase factor of TMF.

Considering that we are not in low magnetic latitudes to envisage any instabilities, we can take the inverse Fourier transform from relation (14) to obtain $B_{z}$. So, we have:

$B_{z}=\mathcal{F}^{-1}\left( \mathcal{F}\left( \psi_{z} \right)\mathcal{F}\left( \mathrm{TMF} \right) \right)$ (15)

The relationship between ${\mathcal{F(}B}_{zz})$ and $\mathcal{F}\left( B_{z} \right)$ is [7]:

$$\mathcal{F}\left( \frac{\partial}{\partial z}B_{z} \right)=|k\mathcal{| F}\left( B_{z} \right)$$

$B_{zz}= \mathcal{F}^{-1} (\left| k \right|\mathcal{F}\left( B_{z} \right))$ (16)

**Reduction to the pole transformation**

In the case of a positive gravity anomaly, the peak value occurs over the mass concentration. This is not true for magnetic anomalies when the magnetization and induced field are not vertical. Reduction to the pole transformation (RTP) gives us the magnetic anomaly data as if it lied in the magnetic pole, thereby the horizontal coordinates of the maximum signal coincides with the horizontal location of the magnetic body.

The relation between Fourier transforms of RTP field and the field in any other place on the Earth is [8]:

$\mathcal{F}\left( \mathrm{RTP} \right)\mathcal{= F}\left( \psi_{\mathrm{RTP}} \right)\mathcal{F}\left( \mathrm{TMF} \right)$ (17)

where $\mathcal{F}\left( \psi_{\mathrm{RTP}} \right)$and $\mathcal{F}\left( \mathrm{TMF} \right)$ are the Fourier transformation of RTP operator and TMF, respectively.

$$\mathcal{F}\left( \psi_{\mathrm{RTP}} \right)=\frac{1}{\theta_{m}\theta_{f}}$$

in which $\theta_{m}=m_{z}+i\frac{m_{x}k_{x}+m_{y}k_{x}}{|k|}$ and $\theta_{f}=f_{z}+i\frac{f_{x}k_{x}+f_{y}k_{x}}{|k|}$. *θ_m_* is phase factor of magnetization, *m*, and *θ_f_* is phase factor of TMF. Taking inverse Fourier transform:

$\mathrm{RTP}=\mathcal{F}^{-1}\left( \mathcal{F}\left( \psi_{\mathrm{RTP}} \right)\mathcal{F}\left( \mathrm{TMF} \right) \right)$ (18)

**Logistic Total Horizontal Gradient**

Logistic Total Horizontal Gradient (LTHG) is a filter to mark the boundaries of geological structures at different depths. It is more effective than TA at delineation of sharp boundaries, where the magnetization direction changes. LTHG is defined as follows [9]:

$LTHG=\left[ 1+exp\left( \frac{\left( \frac{\partial}{\partial z}\left( THG \right) \right)}{\sqrt{\left( \frac{\partial}{\partial x}\left( THG \right) \right)^{2}+\left( \frac{\partial}{\partial y}\left( THG \right) \right)^{2}}} \right) \right]^{-\propto}$ (19)

Where $THG=\sqrt{\left( \frac{\partial}{\partial x}\left( B_{z} \right) \right)^{2}+\left( \frac{\partial}{\partial y}\left( B_{z} \right) \right)^{2}},$and $2\leq\propto\leq10.$

**References**

1. Heiskanen, W. A. & Moritz, H. Physical geodesy. *Bull. Géodésique* **86**, 491–492, https://doi.org/10.1007/BF02525647 (1967).
2. Zengerer, M. An Overview of Tensors, gradient and invariant products in imaging and qualitative interpretation. *ASEG Extended Abstracts* **1**, 1–8. https://doi.org/10.1071/ASEG2018abM3_4E (2018).
3. Pedersen, L. B. & Rasmussen, T. M. The gradient tensor of potential field anomalies: Some implications on data collection and data processing of maps. *Geophysics* **55**, 1558–1566. https://doi.org/10.1190/1.1442807 (1990).
4. Beiki, M. & Pedersen, L. B. Eigenvector analysis of gravity gradient tensor to locate geologic bodies. *Geophysics* **75**, I37–I49. <https://doi.org/10.1190/1.3484098> (2010).
5. Klokočník, J., Kalvoda, J., Kostelecký, J., Eppelbaum, L. V. & Bezděk, A. Gravity disturbances, Marussi tensor, invariants and other functions of the geopotential represented by EGM 2008. *J. Earth Sci. Res.* **2**, 88–101 (2014).
6. Miller, H. G. & Singh, V. Potential Field Tilt – a new concept for location of potential field sources. *J. Appl. Geophys.* **32**, 213–217. https://doi.org/10.1016/0926-9851(94)90022-1 (1994).
7. Nabighian, M. N. Toward a three-dimensional automatic interpretation of potential field data via generalized Hilbert transforms: Fundamental relations. *Geophysics* **49**, 780–786. https://doi.org/10.1190/1.1441706 (1984).
8. Blakely, R. J. *Potential theory in gravity and magnetic applications* (Cambridge University Press, 1996).
9. Pham, L. T., Oksum, E. & Do, T.D. Edge enhancement of potential field data using the logistic function and total horizontal gradient. Acta Geod. Geophys. **54**, 143–155. https://doi.org/10.1007/s40328-019-00248-6 (2019).

**Appendix B**

**Gravity and magnetic signatures of impact craters**

The general gravity and magnetic signatures of impact craters are typically characterized by a high gravity and magnetic annular anomalies encircling a central low. The high anomalies are primarily owing to the uplifted crystalline rim wall rocks and center of the cavity (due to elastic response of the floor in complex craters). In contrast, the interior low anomalies could be due to low density of post-impact sediments and autochthonous breccias in the cavity, and fracturing beneath the floor (in the case of gravity anomaly), and weak magnetization of the impact breccias (in case of magnetic anomaly) [1]. Magnetic parameters of the impact sites are more variable than the gravity in general [2].

Specific factors in determining the final gravity parameters are (i) the size and morphology of the structure, (ii) the density contrast between impact related breccias and target rocks, and (iii) the depth of the structure [1]. Additionally, for small craters supported by lithospheric strength, the short wavelength free air anomaly correlates well with topography. Examples of craters demonstrating a gravity low are: Ries crater in Germany [3] and Siljan crater, Sweden [4]. Depending on the post impact infilling sediments, the low Bouguer anomaly (BA) may turn to a high where some denser material fills the crater and induces a positive anomaly. An example of this type is Sudbury [5]. In a complex crater with central uplift and one or more elevated rings, BA is typically positive over the uplift and the rings. This is because of the higher density of structures uplifted due to the impact (e.g., Marquez Dome, USA [6]). The free air anomaly (FA) has a more stable behavior compared to BA. The excess of mass as well as larger density in the elevated parts (rims and central uplift), usually yield a positive FA. On the contrary, the lack of mass in the interiors of the cavity in conjunction with lower density gives a low FA. It is well documented that regional tectonics, erosion, cooling with associated hydrothermal processes, burial under post-impact sedimentary materials, or being targeted by other projectiles could degrade the gravity and magnetic features of a crater [7].

The shock waves generated in the impact process crush and close the pores of the target rocks,
evaporate the pore water and transfer energy into the target rock, a portion of which turns to heat. This heat melts and vaporizes a part of the target material. The rarefaction and shock waves are the sources of fracturing in the rocks underneath the crater’s floor. The former creates a strong tensile fracturing phase beneath the crater, whereas the latter functions on a compression mode resulting in fracturing especially when the compressive force exceeds the Hugoniot elastic limit [8]. Both processes generate new fracture surfaces and thus cause significant increase in porosity.

Shock metamorphism, shatter cones, planar deformation features, and diaplectic glasses also result in lower overall densities [9,10]. By contrast, the appearance of high-pressure polymorphs causes localized increase in the density, which, however, is insignificant compared to the effect of density decrease by fracturing [9]. Accordingly, the result is a reduced material density caused by the crater formation process, relative to its unshocked surroundings. As the wave travels through the target medium, the energy of the wave declines, thereby decreasing the dimensions and number of the fractures. The fracturing extends beyond the crater’s rim, but its reducing effect on density is so small that it does not significantly affect the gravity variation [11]. Generally, the density of melt sheets is larger than the density of allochthonous breccias, and these two are smaller than the unshocked rock materials away from the crater center [12,13]. At a certain depth (~8 km), the lithostatic pressure closes the fractures and pores of the underlying rocks [14], and the impact related density alterations gradually disappear. That is why in craters with diameters less than 30 km, the negative BA inside the cavity is proportional to the diameter, while in larger craters, the negative quantity reaches a plateau of -30 mGal [2].

The magnetic anomaly is composed of the remanent and induced components of the rock magnetization. The elements like (i) size and shape of the structure, (ii) local geomagnetic field, (iii) direction and amplitude of magnetization of the impactites, and (iv) the altitude in which the data is measured are all effective in the magnetic response of an impact structure [1]. Like its gravity counterpart, a low magnetic anomaly is typically visible over the crater [2], but it is uncorrelated with diameter as shock demagnetization can extend far beyond the actual crater. This low magnetic signature is not the only magnetic characteristic of an impact structure. Several complex craters display high amplitude, shallow (high frequency) anomalies over the central uplift (e.g., Manicouagan crater), that are likely caused by the induced component of hydrothermal alterations within the fractured central uplift [15]. Other craters have slight positive anomalies over their centers or even no specific impact related magnetic signature [16]. While such anomalies may exist over those craters, they may also indicate traces of electric currents that resulted from the plasma separation effects [17,18].

The observed magnetic anomalies of the impact craters are subdued due to plasma shielding effects [18]. However, the shock is responsible for magnetic hardening, which means an increase in magnetic coercivity due to shock modification/recrystallization of the crystal lattices of magnetic carriers, locking the domain walls, precipitation of new magnetic phases, and thus modifying the rock magnetic signature. The impact hardening is indicated by the Koenigsberger ratio, being >30 on impact melts and breccias of the Vredefort crater [19], ~10 for the Mien crater [20], and >10 for the Haughton crater [21]. While the remanence is more stable, it is generally significantly lower than if the same rock was magnetized in the cooling magnetic field (thermal remanent magnetization) due to shielding of the geomagnetic field at the time of impact [18].

When ferrimagnetic phases are present in the hosting rocks or if they are created by oxidation, the amount of magnetization is large [22]. Magnetite, hematite and pyrrhotite are the main carriers of magnetization of the target rocks [23]. On the other hand, impacting meteorites contain iron and nickel containing minerals with variable magnetic properties. However, given the minuscule volumetric percentage of the meteorite compared to the size of the crater, the magnetic properties of meteorites may not significantly contribute to the magnetic response of the impact area (e.g., Lappajärvi [24]).

The magnetic behavior of rocks and minerals is influenced by the shock wave. Depending on the intensity of the geomagnetic field, the magnitude of the shock wave and the mineral magnetic hardness, the natural remanent magnetization of rock can be eliminated (shock demagnetization) or overprinted by shock remanent magnetization (SRM) [25]. SRM, however, resides in low coercivity grains and therefore does not contribute significantly to the magnetic remanence. SRM is usually parallel to the ambient magnetic field [26]. As the shock wave rises (up to 35 GPa), the coercive force of the magnetic minerals increases. Consequently, magnetic shock hardening develops in rock [27,28].

Because of the upward motion of the hot, compressed, and dense materials in the elevated parts of the impact structure (the central uplift and rims), as well as the smaller distance of measurement in these areas, the magnetic response could be enhanced. This is boosted when the stronger magnetic crystalline basement underlies less magnetic sedimentary layers [21].

As with the gravity, the magnetic signature can also be affected, altered or eliminated by post-impact processes. For example, oxidation may change magnetite to hematite and decrease the magnetic anomaly [29], or chemical remanent magnetization may be generated due to the chemical alteration of minerals [30].

**References**

1. Plado, J. Gravity and magnetic signatures of meteorite impact structures. Ph.D. Thesis (University of Tartu, Estonia) <https://core.ac.uk/download/pdf/79106852.pdf>  (2000).
2. Pilkington, М. & Grieve, R. A. F. The geophysical signature of terrestrial impact craters. *Rev. Geophys.* **30**, 161–181. <https://doi.org/10.1029/92RG00192> (1992).
3. Pohl, J., Stöffler, D., Gall, H. & Ernston, K. The Ries impact crater in *Impact and Explosion Cratering* (eds. Roddy, D. J., Pepin, R. O. & Merrill, R. B.) 343–404 (Pergamon Press, 1977).
4. Dyrelius, D. The gravity field of the Siljan ring structure in *Deep Drilling in Crystalline Bedrock, Vol. 1: The deep gas drilling in the Siljan impact structure, Sweden and astroblemes* (eds. Bodén, A. & Eriksson, K. G.) 85–94 (Springer Verlag, 1988).
5. McGrath, P. H. & Broome, H. J. A gravity model for the Sudbury Structure along the Lithoprobe seismic line. *Geophys. Res. Lett.* **21**, 955–958. <https://doi.org/10.1029/93GL02247> (1994).
6. Wong, A. M., Reid, A. M. & Hall, S. A. Characterization of the Marquez Dome buried impact crater using gravity and magnetic data. *Lunar Planet. Sci. C.* **XXIV**, 1533–1534 (1993).
7. Plado, J. & Puura, V. Gravity and magnetic signatures of differently eroded buried impact craters, calculated from Kärdla crater. *Ann. Geophys.* **13** (suppl. III), C741 (1995).
8. Melosh, H. J. *Impact Cratering. A Geologic Process* (Oxford University Press, 1989).
9. Koeberl, C. Impact cratering: The mineralogical and geochemical evidence. *Okla. Geol. Surv. Circ.* **100**, 30–54 (1997).
10. Stöffler, D. Deformation and transformation of rock-forming minerals by natural and experimental processes: 2. Physical properties of shocked minerals. *Fortschr. Mineral.* **51**, 256-289 (1974).
11. Gurov, E. P. & Gurova, E. P. Some regularities of the areal spreading of fractures around Elgygytgyn impact crater. *Lunar Planet. Sci. C.* **XIII**, 291–292 (1982).
12. Dabizha A. I. & Feldman V. I. The geophysical properties of some astroblemes in the USSR. *Meteoritika* **40**, 91–101 (1982) (in Russian).
13. Elo, S., Jokinen, T. & Soininen, H. Geophysical investigations of the Lake Lappajärvi impact structure, western Finland. *Tectonophysics* **216**, 99–109. <https://doi.org/10.1016/0040-1951(92)90158-3> (1992).
14. Perrier, R. & Quiblier, J. Thickness changes in sedimentary layers during compaction history: Methods for quantitative evaluation. *Am. Assoc. Petr. Geol. B.* **58**, 507–528. <https://doi.org/10.1306/83D9142A-16C7-11D7-8645000102C1865D> (1974).
15. Coles, R. L. & Clark, J. F. The central magnetic anomaly, Manicouagan Structure, Quebec. *J. Geophys. Res.* **83**, 2805–2808. <https://doi.org/10.1029/JB083iB06p02805> (1978).
16. Pilkington, M., Jansa, L. F., & Grieve, R. A. F. Geophysical studies of the Montagnais impact crater, Canada. *Meteoritics* **30**, 446–450. <https://doi.org/10.1111/j.1945-5100.1995.tb01151.x> (1995).
17. Crawford, D. A. Simulations of magnetic fields produced by asteroid impact: Possible implications for planetary paleomagnetism. *Int. J. Impact Eng.* **137**, 103464. <https://doi.org/10.1016/j.ijimpeng.2019.103464> (2020).
18. Kletetschka, G., Kavkova, R. & Ucar, H. Plasma shielding removes prior magnetization record from impacted rocks near Santa Fe, New Mexico. *Sci. Rep.* **11**, 22466. <https://doi.org/10.1038/s41598-021-01451-8> (2021).
19. Hart, R. J., Hargraves, R. B., Andreoli, M. A. G., Tredoux, M. & Doucouré, C. M. Magnetic anomaly near the center of the Vredefort structure: Implications for impact-related magnetic signatures. *Geology* **23**, 277–280. <https://doi.org/10.1130/0091-7613(1995)023%3C0277:MANTCO%3E2.3.CO;2> (1995).
20. Stanfors, R. The Mien structure – A cryptoexplosion formation in the Fennoscandian basement. Ph.D. Thesis (Lund University, Sweden, 1973).
21. Pohl, J., Eckstaller, A. & Robertson, P. B. Gravity and magnetic investigations in the Haughton impact structure, Devon Island, Canada. *Meteoritics* **23**, 235–238. <https://doi.org/10.1111/j.1945-5100.1988.tb01286.x> (1988).
22. Henkel, H. Geophysical aspects of meteorite impact craters in eroded shield environment, with special emphasis on electric resistivity. *Tectonophysics* **216**, 63–89. <https://doi.org/10.1016/0040-1951(92)90156-Z> (1992).
23. Butler, R. F. *Paleomagnetism: Magnetic Domains to Geologic Terranes* (Blackwell Scientific Publications, 1992).
24. Fregerslev, S. & Carstens, H. Fe-Ni metal in impact melt rocks of Lake Lappajärvi, Finland. *Contrib. Mineral. Petr.* **55**, 255–263. <https://doi.org/10.1007/BF00371336> (1976).
25. Pohl, J., Bleil, U. & Hornemann, U. Shock magnetization and demagnetization of basalt by transient stress up to 10 kbar. *J. Geophys.* **41**, 23–41. (1975).
26. Halls, H. C. The Slate Islands meteorite impact site: A study of shock remanent magnetization. *Geophys. J. Int.* **59**, 553–591. <https://doi.org/10.1111/j.1365-246X.1979.tb02573.x> (1979).
27. Cisowski, S. M. *et al.* Magnetic effects of shock and their implications for lunar magnetism (II). *P. Lunar Sci. C.* **7**, 3299–3320 (1976).
28. Pesonen, L. J., Deutsch, A., Hornemann, U. & Langenhorst, F. Magnetic properties of diabase samples shocked experimentally in the 4.5 to 35 GPa range. *Lunar Planet. Sci. C.* **XXVIII**, 1087–1088 (1997).
29. Elming, S. Å. & Bylund, G. Paleomagnetism and the Siljan impact structure, central Sweden. *Geophys. J. Int.* **105**, 757–770. <https://doi.org/10.1080/11035899209453472> (1991).
30. Steiner, M. & Shoemaker, E. Two-polarity magnetization of the Manson impact breccias. *Lunar Planet. Sci. C.* **XXIV**, 1347–1348 (1993).
